# Diagnostic performance of 3T stress magnetic resonance myocardial perfusion imaging (MRMPI) using 32-channel cardiac coil in patients with coronary artery disease

**DOI:** 10.1186/1532-429X-13-S1-P95

**Published:** 2011-02-02

**Authors:** Chun-Ho Yun, Jui-Peng Tsai, Tien-Yu Wu, Cheng-Ting Tsai, Ricardo C Cury

**Affiliations:** 1 Mackay Memorial Hospital, Taipei, Taiwan; 2 Baptist Cardiac Vascular Institute, Miami, FL, USA

## Background/aim

With newly available 32-channel cardiac coil, 3 tesla MRI system provides increased signal-to-noise radio (SNR), reduced imaging time and improved spatial resolution. This study sought to determine the diagnostic performance of 3T stress MRMPI with 32-channel cardiac coil in detecting clinical relevant coronary artery stenosis in comparison with invasive coronary angiography (ICA).

## Methods

Forty four patients (29 men; mean age: 61year) who were scheduled for ICA underwent stress MRMPI with 3T MRI (Achieva 3T, Philips) with a 32-channel cardiac receiver coil ( Invivo, Gainesville, FL). The total amount of contrast medium (Multihance, Bracco) was 0.15mmole/kg with injection rate at 4ml/s. The MR protocol included gadolinium-enhanced stress first-pass perfusion (0.56mg/kg, dipyridamole), rest perfusion, and delayed enhancement (DE). Ischemia was defined as (1) a territory with perfusion defect at stress study but no DE, (2) territory with DE but additional peri-infarcted perfusion defect at stress study. Diagnostic performance was determined on a per-patient basis. Quantitative CA served as the reference standard.

## Results

Coronary angiography depicted significant stenosis (≥70% stenosis at epicardial coronary artery) in 22 of 44 patients (50%). No complications were observed during Stress perfusion 3.0 tesla MRI and dipyridamole infusion. For detection of significant coronary stenosis, 3T stress MRMPI provided sensitivity: 0.91(0.75-1.00), specificity: 0.86(0.72-1.00), positive predictive value: 0.87(0.74-1.00) and negative predictive value: 0.91(0.748-1.00). The overall diagnostic accuracy was: 0.88.

## Conclusion

Our study showed that 3T stress MRMPI using 32-channel cardiac coil demonstrated high diagnostic accuracy for detection of significant stenosis, having QCA as the reference standard. Further investigation is needed to assess if advances in technology and improvement in diagnostic accuracy for CAD detection will translate in better patient care and outcomes.

**Figure 1:**
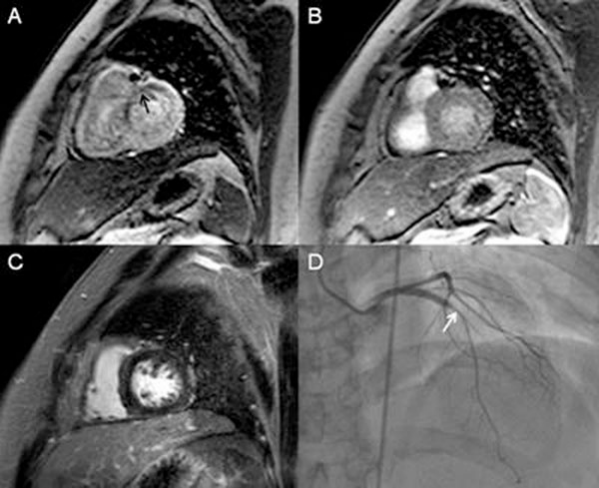
**Image of a patient demonstrating stress-inducible subendocardial perfusion defect (black arrow) at basal anterior and antero-septal walls during stress study (A); but not during rest study (B).** No delayed enhancement is present in the anterior wall.(C). Invasive coronary angiography shows high grade edge instent restenosis (white arrow) of LAD-p (D)

